# Personal

**Published:** 1905-08

**Authors:** 


### PERSONAL.

Dr. Edward N. Brush, physician in chief and superintendent of the Sheppard and Enoch Pratt Hospital, Towson, Md., chose for the subject of his address as retiring president of the medical and chirurgical faculty of Maryland, delivered April 25, 1905, "The Physician as a Citizen." It is a scholarly paper and handles the subject in a masterful manner.

Dr. G. Frank Lydston, of Chicago, has been elected a fellow of the London Society of Authors, an honor which has come to very few American writers. This is a graceful recognition on the part of the Londoners of a very brilliant author who, in addition to being remarkably versatile, is intensely interesting.

---

Dr. Chauncey Pelton Smith, of this city, offers physician's offices for rent at "The Raleigh," 354 Franklin street, near Tupper, Buffalo, N. Y. The suite consists of waiting and consulting rooms, two bedrooms and private bath. Enquire of Dr. Smith on the premises, or of Sherman S. Jewett, D. S. Morgan building.

---

Dr. J. H. Pryor, superintendent of the New York State Hospital for Incipient Tuberculosis at Raybrook, N. Y., has resigned. He will reside at Saranac Lake and resume the practice of medicine.

---

Dr. Charles W. Pillsbury, of Chicago, is visiting Buffalo for a few weeks in the interest of Armour & Company. Dr. Pillsbury has many friends in this city who look forward to his annual visits with pleasure.

---

Dr. Harry H. Ebberts, a graduate of the University of Buffalo, 1904, has gone to Europe to continue his medical studies.

---

Dr. James Wright Putnam, of Buffalo, attended the recent meeting of the American Medical Association at Portland. Dr. Putnam was accompanied by his son Jack.

---

Dr. George F. Cott, of Buffalo, accompanied by Mrs. Cott, attended the meeting of the American Medical Association at Portland, in July. Dr. Cott served as a member of the house of delegates.

---

Dr. Dewitt C. Greene, of Buffalo, who, in company with Mrs. Greene and Senator Henry W. Hill, has been spending the last three months in Europe, has recently returned and resumed his professional work.

---

Dr. Mary O'Malley, a graduate of Niagara University, 1897, who has been serving as woman physician at the Binghamton state hospital since 1898, has recently been appointed to a similar position in the government hospital for the insane at Washington, D. C.

---

Dr. Henry Lapp, of Clarence, entertained the Medical Union of Buffalo during its regular monthly meeting for July.

---

Dr. Melvin Page Burnham, of New York, has been appointed acting superintendent of the New York State Hospital for Incipient Tuberculosis at Raybrook, vice Dr. John H. Pryor, resigned.

